# Perceived Changes in Anxiety Symptom Burden During Treatment with *Bryophyllum pinnatum*: A Prospective, Single-Arm Study

**DOI:** 10.3390/ph17111423

**Published:** 2024-10-24

**Authors:** Tiffany Huber, Daniel Krüerke, Timotheus Haeck, Markus Weber, Matthias Kröz, Markus Schlemmer, Ana Paula Simões-Wüst

**Affiliations:** 1 Clinical Research Department, Klinik Arlesheim, 4144 Arlesheim, Switzerland; tiffany.huber@klinik-arlesheim.ch (T.H.); daniel.krueerke@klinik-arlesheim.ch (D.K.); timotheus.haeck@klinik-arlesheim.ch (T.H.); matthias.kroez@klinik-arlesheim.ch (M.K.); 2 Clinical Research Department, Society for Cancer Research, 4144 Arlesheim, Switzerland; 3 Department of Psychiatry and Psychosomatics, Klinik Arlesheim, 4144 Arlesheim, Switzerland; markus.weber@klinik-arlesheim.ch (M.W.); markus.schlemmer@klinik-arlesheim.ch (M.S.); 4 Sleep Medicine, Klinik Arlesheim, 4144 Arlesheim, Switzerland; 5 Research Institute Havelhöhe, 14089 Berlin, Germany; 6 Institute for Integrative Medicine, University Witten/Herdecke, 58455 Witten, Germany; 7 Department of Obstetrics, University Hospital Zurich, University of Zurich, 8006 Zurich, Switzerland

**Keywords:** *Bryophyllum pinnatum*, *Kalanchoe pinnata*, Anthroposophic Medicine, mental disorders, anxiety, depression

## Abstract

Background/Objectives: *Bryophyllum* spp. preparations are widely used in Anthroposophic Medicine, most often for mental and behavioral disorders, including anxiety. Studies in animals revealed various anxiolytic and neurosedative effects for *Bryophyllum pinnatum*. We set out to investigate the effectiveness of Bryophyllum 50% chewable tablets, a product registered without indication by means of notification in Switzerland, in the treatment of anxiety symptoms in psychiatric and psychosomatic patients. Methods: A total of 99 patients with anxiety symptoms were recruited from the waiting list for an inpatient stay at the hospital “Klinik Arlesheim”, Department of Psychiatry and Psychosomatics; of these, 54 completed the study and returned fully completed questionnaires. Patients were treated with Bryophyllum 50% chewable tablets (350 mg tablets, made from leaf press juice, 3 × 2 per day; Weleda AG Arlesheim, Switzerland) and filled out questionnaires at baseline and after two and three weeks of tablet intake. The primary endpoint of the study was the change in anxiety symptoms measured with the Beck Anxiety Inventory (BAI). Results: A clinically relevant decrease in BAI score from baseline (27.4 ± 12.0) to after two (22.4 ± 12.1; *p* < 0.001) and three (20.6 ± 12.9; *p* < 0.001) weeks of treatment was observed. Additional improvements were observed in the secondary endpoints (including in depression, sleep quality, and stress); tolerability and compliance were very good. Conclusions: The results suggest that Bryophyllum 50% chewable tablets have beneficial effects on anxiety-related symptoms. Since the study design does not allow us to conclude causality between treatment and observed improvements, a randomized clinical trial is urgently needed.

## 1. Introduction

Anxiety disorders are among the world’s most common mental disorders, with a lifetime prevalence of 4%. Furthermore, the number of people affected by anxiety disorders rose by 55% between 1990 and 2019 [1]. Current research on Switzerland, a country with one of the highest prevalence rates, indicates that the prevalence of anxiety in young adults is 13%, with women more often affected than men [2]. Anxiety disorders peaked in the population aged 35–96 during the first COVID-19 pandemic wave in February 2021 [3]. Excessive anxiety is linked to significant mental and physical impairments and may result in a dramatic reduction in quality of life and socioeconomic well-being, which in turn is a promoting factor for anxiety disorders [4]. People with anxiety symptoms suffer from worrying or fear impending danger and sympathetic autonomic overactivity, such as sweating, heart palpitations, and indigestion. Anxiety is often accompanied by depression, stress, and poor sleep quality [5,6].

There are several pharmacotherapy options for the treatment of anxiety disorders—such as selective serotonin reuptake inhibitors (SSRIs), selective serotonin–norepinephrine reuptake inhibitors (SSNRIs), other antidepressants, and benzodiazepines—with varying degrees of effectiveness for each option [7,8]. Most of these medications can produce side effects that interfere with the patient’s quality of life and eventually lead to low compliance. These side effects may include weight gain, nausea, headaches, insomnia, and sexual dysfunction; moreover, development of treatment resistance and situations of addiction/dependence (particularly important in the case of prolonged use of benzodiazepines [9]) might occur. In recent decades, research and use of phytomedicines to treat anxiety have increased considerably [10,11]. Several phytomedicines have been shown to have anxiolytic effects through main neurotransmitter pathways, such as γ-aminobutyric acid (GABA), acetylcholine (ACh), glutamate, serotonin (5-HT), dopamine (DA), and norepinephrine (NE) [12]. For example, a randomized controlled trial (RCT) showed anxiolytic effects of Aloysia polystachya (Griseb.) Moldenke (Verbenaceae) after four and eight weeks in adults with self-reported anxiety symptoms [13]. Another RCT reported reduced anxiety symptoms after ten weeks’ treatment with a lavender oil preparation in generalized anxiety disorder (GAD) [14]. Phytomedicines might contribute to the avoidance of or at least lowering doses of synthetic medications, i.e., reducing their side effects. Moreover, phytomedicines are often easily accessible products, which might facilitate self-management (in contrast to, e.g., psychotherapy, and particularly important due to the highly persistent stigma against taking psychoactive medications) [15].

*Bryophyllum pinnatum* (Lam.) Oken (Crassulaceae) is a succulent plant native to Madagascar. It grows well in tropical and subtropical regions of America, Africa, India, Asia, and Australia, where it is used due to its analgesic, antipyretic, gastro-protective, anti-inflammatory, antiseptic, antiallergic, antianaphylactic, antileishmanial, antiulcer, wound healing, and sedative properties [16,17,18,19,20,21,22]. In Europe, *Bryophyllum* spp. were first used at the beginning of the 20th century for hyperactivity disorders in Anthroposophic Medicine (AM), a multimodal medical system integrating modern conventional medicine and anthroposophic principles of health and disease founded by Rudolf Steiner and Ita Wegman [23]. The study of AM is always in addition to the standard medical curriculum. However, the certification requirements to become an anthroposophic physician are defined and regulated on national levels. According to the federation of anthroposophic-oriented physicians in Switzerland (VAOAS), the acquisition of the certificate of competence, the “physician for Anthroposophic extended Medicine VAOAS/SIWF”, requires a medical specialist license.

Initially introduced because of their calming and sedative effects, *Bryophyllum* spp. preparations are widely used in AM for the treatment of mental and behavioral disorders, such as anxiety, sleep disorders, and depressive symptoms. This was shown in a previous study where anthroposophic physicians documented their prescriptions between January 2004 and January 2010 (see [24] and references therein). These indications are also in accordance with the German Commission C, which states that *Bryophyllum* spp. preparations are to be used for (among other things) emergency situations of anxiety or other related mental problems and associated sleep disorders. This commission’s role is to advise the Federal Institute for Medicinal Products and Medical Devices on the approval of Anthroposophic Medicines.

Bryophyllum 50% chewable tablets are produced according to Good Manufacturer Practice (GMP) with *Bryophyllum pinnatum* (BP) leaf press juice from plants cultured in Brazil and are registered at the Swiss Agency for Therapeutic Products (Swissmedic) without indication by means of notification (more information available in Section 4.3). In Switzerland, over the last decade, the tablets have also started to be used in conventional settings [16], mainly in the treatment of preterm labor [25], overactive bladder [26], and restless legs syndrome [27]. Three prospective observational studies revealed positive effects of treatment with Bryophyllum 50% on sleep quality during pregnancy (*n* = 49, treatment for two weeks) [28] or among cancer patients (*n* = 20, three weeks’ treatment) [29] and patients suffering from nocturia (*n* = 49, three weeks’ treatment) [30]. In the two latter cases, several significant improvements in sleep quality as assessed with the Pittsburgh Sleep Quality Index (PSQI) were observed. Finally, a recent survey among physicians and care personnel carried out at the Klinik Arlesheim revealed good perceived reactions to *Bryophyllum* spp. preparations in the treatment of anxiety [31]. All studies performed so far suggest very good safety/tolerability of BP preparations. To our knowledge, however, no phase I studies have been performed with BP preparations. In our previous work, the cytotoxic effects of BP leaf press juice have been investigated at the cellular level. Also in this in vitro experimental set-up, no signs of toxicity were found, as it did not affect cell viability even at concentrations far higher than those needed to observe effects in various models [32].

The use of preparations produced from BP in human mental and behavioral disorders is supported by results from animal models in mice, rats, and zebrafish, where they have shown CNS-depressant as well as anxiolytic and neurosedative effects. In particular, observed improvements in sleep disorders were corroborated by rodent experiments showing that different fractions of the leaf extract of BP can prolong pentobarbitone-induced sleeping time [33,34,35]. Moreover, BP was shown to exert changes in anxiety-like behavior in zebrafish, suggesting psychoactive and anxiolytic effects [36]. Ongoing investigations point towards an activation of GABA receptors as a mechanism of action for BP extracts [37].

Various phytochemical studies of BP have led to the identification of flavonoids and bufadienolides [38,39], as well as of triterpenes, phytosterols, fatty acids, and minerals [18]. Plant flavonoids in general—polyphenolic compounds in which two benzene rings are bound together with a heterocyclic pyran or a pyrrole ring—are biologically active. These naturally occurring compounds have been described as having anti-inflammatory, anticarcinogenic, antiallergic, and neuroprotective properties [22,40,41,42,43,44]. The HPLC-UV/ESI-MS analysis of a methanol extract of the press juice of fresh or fresh-frozen BP leaves revealed flavonoid glycosides as characteristic constituents [45]. Two phenolic glucosides, namely, syringic acid β-D-glucopyranosyl ester and 4′-O-β-D-glucopyranosyl-cis-p-coumaric acid, as well as nine glycosides of the flavonoids kaempferol, quercetin, myricetin, acacetin, and diosmetin, were identified by ^1^H and two-dimensional nuclear magnetic resonance spectroscopy analysis. Interestingly, the existence of two of these flavonoids, namely, quercetin 3-O-α-L-arabinopyranosyl-(1→2)-α-L-rhamnopyranoside 7-O-β-D-glucopyranoside and myricetin 3-O-α-L-arabinopyranosyl-(1→2)-α-L-rhamnopyranoside, was until then unknown in any plant extracts. Furthermore, the following four bufadienolides were detected: bersaldegenin-1-acetate, bryophyllin A, bersaldegenin-3-acetate, and bersaldegenin-1,3,5-orthoacetate [45]. A quantitative assay revealed that leaf press juice (of plants cultured in Brazil) comprised 0.9 to 1.2 mg/100 mL of bufadienolides [46]. Some of the bufadienolides present in Bryophyllum species are thought to be responsible for their central sedative properties [47,48].

Despite the promising pharmacological evidence, to date, there have been no prospective clinical studies addressing the effectiveness of *Bryophyllum* spp. preparations in the treatment of anxiety symptoms. The main aim of the present prospective study was to find out if patients suffering from anxiety symptoms perceive improvements in their symptoms during treatment with Bryophyllum 50% chewable tablets according to self-reported inventories. Associated depression, reduced sleep quality, and stress were assessed as well. Finally, health-related quality of life (mental and physical) and internal coherence were investigated. Here, averaged data from the entire patient population were analyzed; in a separate work, subgroup analyses will be performed to find out which patients might benefit most from the treatment (manuscript in preparation). Taken together, the results will enable planning randomized trials on the use of Bryophyllum 50% chewable tablets in the treatment of anxiety symptoms.

## 2. Results

### 2.1. Population Characteristics

Between June 2021 and November 2023, 99 patients were recruited among patients from the waiting list for an inpatient stay at the Department of Psychiatry and Psychosomatics at the Klinik Arlesheim, Switzerland. Of these, 56 patients completed the study and returned the part 1 (baseline) and part 2 (after two weeks’ treatment) questionnaires. Two patients (out of these fifty-six) had to be excluded from the analysis because the first or second BAI questionnaire contained more than three missing values. Therefore, the main analysis (comparison of the results at baseline and after two weeks’ treatment) could be performed with 54 patients. Five patients had one item missing from the BAI questionnaire at baseline, three patients had a missing item in the BAI after two weeks, and two patients had a missing item in the BAI after three weeks. Forty-eight patients also sent in the BAI questionnaire after three weeks’ treatment; in the case of the six patients who did not, the missing values were replaced by the values after two weeks (last observation carried forward, LOCF).

Detailed demographic characteristics are shown in Table 1. In brief, the mean age and BMI of the study patients were 51.0 years (*n* = 53) and 23.5 (*n* = 52), respectively. The majority of the participants identified as women (78.8%), had a higher or tertiary education (both 52.9%), lived alone (approximately 50%), and participated in some sports (59.3%). Some participants reported nicotine consumption.

Patients’ health-related data are shown in Table 2. Among the health disorders mentioned in the questionnaires, some patients reported having diabetes and high blood pressure. In addition, 32 allergies were reported by 19 patients. In the following, these are listed, even if in some cases a hypersensitization/intolerance appears likely: pollinosis (*n* = 9), cats (*n* = 3), house dust (*n* = 3), acetylsalicylic acid (*n* = 2), hazelnut (*n* = 2), red wine (*n* = 1), cheese (*n* = 1), amoxicillin with clavulanic acid (*n* = 1), metamizol (*n* = 1), phenylendiamin (*n* = 1), stone and pomaceous fruits (*n* = 1), soy (*n* = 1), ficus (*n* = 1), nickel (*n* = 1), copper (*n* = 1), fragrances (*n* = 1), latex (*n* = 1), and sulphite (*n* = 1). Furthermore, a few cases of milk (*n* = 1) and lactose (*n* = 1) intolerances and celiac disease (*n* = 1) were mentioned. As was to be expected from the study inclusion criteria, all patients reported one or more mental disorders and/or symptoms. More than three-quarters of the patients reported having sleep problems and anxiety symptoms, as well as suffering from anxiety and depression disorders. A posterior review of the patients’ clinical histories revealed that, in total, 45 patients were suffering from depressive disorders as a main diagnosis, with a variety of additional mental disorders/conditions mentioned as well.

At recruitment and in accordance with the reported mental disorders, the patients were taking a variety of medications; note that according to the inclusion criteria (described in Section 4.2), patients were excluded only if dose increases or introduction of new (conventional) anxiolytics or antidepressants were planned. Medications were reported by the patients in free text fields mostly by trade names, and in some cases also by the active substance. All medications are listed in the text below. For an overview of the types of active substances used by the patients, see Table 3. Antidepressant, anxiety-reducing, and soporific medications were grouped according to their characteristics as declared in the Swiss Pharmaceutical Compendium (in German “Arzneimittel Kompendium der Schweiz^®^”; https://compendium.ch/) and taking into consideration that some medications have more than one characteristic. The medications taken at recruitment comprise in most cases antidepressants, anxiolytics, and hypnotics; a few medications for physical disorders were mentioned as well.

Synthetic antidepressants being taken included duloxetine (*n* = 2) and venlafaxine (*n* = 3), both of which are selective serotonin–norepinephrine reuptake inhibitors (SSNRIs, *n* = 5); escitalopram (*n* = 6), sertraline (*n* = 3), fluoxetine (*n* = 1), paroxetine (*n* = 1), and citalopram (*n* = 1), which are selective serotonin reuptake inhibitors (SSRIs, *n* = 12); bupropion (*n* = 2), a selective noradrenaline and dopamine reuptake inhibitor (SNDRI, *n* = 2); tradozone (*n* = 8), a serotonin antagonist and reuptake inhibitor (SARI, *n* = 8); trimipramine (*n* = 1), a tricyclic antidepressant (TCA, *n* = 1); and mirtazapine (*n* = 2), a tetracyclic antidepressant (NaSSA, *n* = 2). In addition, several phytomedicines were reported, namely, St. John’s wort (*n* = 6), cannabidiol (*n* = 3), and a mixture of valerian, purple passionflower, lemon balm, and butterbur (*n* = 3).

Reported anxiety-reducing medications were lorazepam (*n* = 5), alprazolam (*n* = 2), pregabalin (*n* = 2), bromazepam (*n* = 1), and temazepam (*n* = 1), all five being classified as anxiolytics, as well as the following antidepressants with anxiety-reducing effects: duloxetine (*n* = 2), venlafaxine (*n* = 3), escitalopram (*n* = 6), citalopram (*n* = 1), sertraline (*n* = 3), paroxetine (*n* = 1), trazodone (*n* = 8), and trimipramine (*n* = 1). Cannabidiol (*n* = 3) and a mixture of valerian, purple passionflower, lemon balm, and butterbur (*n* = 3) were the reported antidepressant phytomedicines.

The following medications with sedative characteristics were reported: trazodone (*n* = 8), mirtazapine (*n* = 2), and trimipramine (*n* = 1) as antidepressants; temazepam (*n* = 1), lorazepam (*n* = 5), and zolpidem (*n* = 2) as anxiolytics; St. John’s wort (*n* = 6), a preparation with valerian roots and hop cones (*n* = 2), avena sativa (*n* = 1), cannabidiol (*n* = 3), a product with valerian, purple passionflower, lemon balm, and butterbur (*n* = 3), and valerian root (*n* = 1) as herbal medications; and quetiapine (*n* = 4) as a neuroleptic.

In addition to the medications, the majority of the patients made use of talking therapies (*n* = 33), and one in four (*n* = 13) made use of therapies from complementary medicine. Talking therapies included psychotherapy, sessions with a psychiatrist, sessions with a psychologist, talking sessions with a general practitioner, talking therapy (without precise information), and psychiatric home care services (Spitex, Switzerland). Therapies from complementary medicine included art therapy, eurythmy therapy, craniosacral therapy, acupuncture, osteopathy, kinesiology, naturopathy, somatic experience, shiatsu, neural therapy, riding therapy, and meditation.

### 2.2. Effectiveness

**Figure 1:**
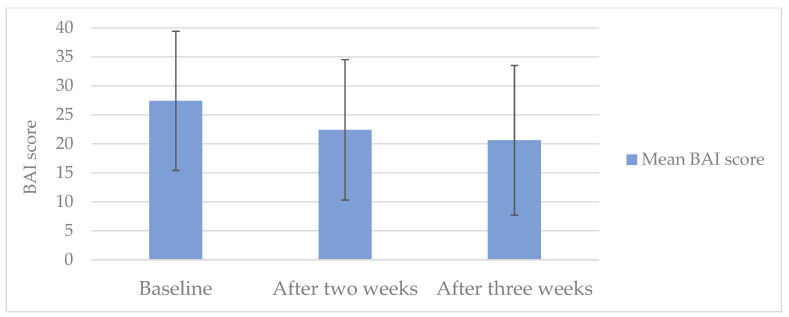
Mean BAI score (*n* = 54) at baseline and after two and three weeks of treatment with Bryophyllum 50% chewable tablets. Error bars indicate SD.

A significant reduction in the anxiety symptom burden assessed with the BAI questionnaire (−5.0 points; *p* < 0.001) was observed between baseline and after two weeks of treatment (main outcome; see Figure 1). The mean BAI score dropped from the category “severe anxiety” at baseline to “moderate anxiety” two weeks later. There was also an improvement according to the BAI score between baseline and after three weeks of treatment.

As shown in Table 4, the decrease in the BAI sum score was accompanied by significant reductions in all four subdomains, subjective, neurophysiologic, autonomic, and panic-related, after both two and three weeks of treatment. The changes in BAI scores correspond to an improvement of 18.2% and 24.8% after two and three weeks of treatment.

**Figure 2:**
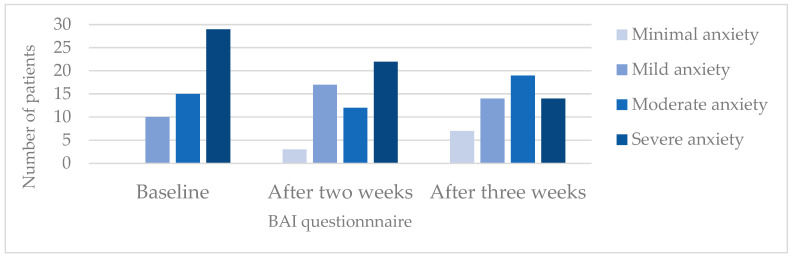
Changes in BAI classification patterns in the course of treatment with Bryophyllum 50% chewable tablets. This graph shows the number of patients according to the four classification groups of the BAI.

As mentioned under Methods (see Section 4.5), BAI allows a classification of the anxiety intensity. In Figure 2, the numbers of patients in the various intensity subgroups at study start and after two and three weeks of treatment are depicted. The data show that the number of patients with severe anxiety decreased during treatment, while the number of patients with minimal and mild anxiety increased.

All secondary outcomes also showed improvements in questionnaire scores between baseline and after two and three weeks of treatment, although with varying intensities. Detailed score values of the GAD-7, PHQ-9, PSS, SF-12, and ICS questionnaires are shown in Table 5. A comparison of the changes in the SF-12 scores for physical and mental health reveals that marked increases were observed in the latter, while only slight improvements were detected in physical health; the higher Cohen’s d value for the mental health score reflects this observation. Additional comparatively higher Cohen’s d values were obtained for BAI, GAD-7, and PHQ-9.

### 2.3. Compliance and Tolerability During the Study and Eventual Hospital Admission at Study End

As shown in Table 6, the average number of tablets the patients took was 118 ± 16 (*n* = 50, maximum 132 tablets). Eleven tablets should have been left after three weeks of treatment. The number of tablets taken was either reported by the patients and coincided with the number of tablets in the returned medication flasks as counted by the study team (*n* = 38) or was solely counted by the patients (*n* = 5, flask not returned to the study team) or was counted only by staff (*n* = 7, information missing in the questionnaire). Thirty-two patients made use of the opportunity to request additional Bryophyllum 50% chewable tablets for the time after the study (patients on the waiting list for longer than three weeks). Most of the patients reported not changing the dosage either after two (*n* = 41) or after three (*n* = 37) weeks of treatment. During the first two weeks of treatment, seven patients reported a dose decrease in other medications (mostly with anxiolytic /antidepressant effects), whereas one reported an increase (of cannabidiol).

In total, 12 patients (out of 54) reported 22 perceived discomforts after two and three weeks, which they thought could be associated with the study medication. Of these 12 patients, 9 noted problems with digestion such as diarrhea (*n* = 2), constipation (*n* = 1), nausea (*n* = 2), undefined intestinal problems (*n* = 1), and some reported experiencing a burning sensation in their stomach (*n* = 3). In addition, three patients reported anxiety- or emotion-related discomforts, two felt dizzy, and two felt tired. Other reported issues included the following: experiencing itching on the back of the hand (*n* = 1), feeling more sensitive to pain (*n* = 1), high blood pressure (*n* = 1), infected oral mucosa (*n* = 1), increased thirst (*n* = 1), and feeling numb legs (*n* = 1). Of these 12 patients perceiving discomforts, 10 patients mentioned one symptom repeatedly.

One patient was hospitalized for cardiac decompensation (NYHA class IV symptoms) in the context of cardiomyopathy and hypertensive heart disease (with previously implanted pacemaker), accompanied by anemia (requiring blood transfusion), chronic obstructive lung disease, and middle kidney insufficiency, which had to be classified as a serious adverse event (SAE). Since there was extensive preexisting internal disease that was not fully known at patient recruitment, a causal relationship with the treatment with Bryophyllum 50% was not assumed.

After study end, 38 of the recruited 54 patients were admitted to the hospital “Klinik Arlesheim”.

## 3. Discussion

Taken together, the results presented here show that anxiety symptoms measured with the BAI in psychiatric and psychosomatic outpatients are significantly reduced after two and three weeks of Bryophyllum 50% intake (2 × 3 chewable tablets per day, with high compliance). Mean BAI scores dropped from the category severe anxiety to moderate anxiety after two and three weeks. Additional marked improvements were observed in general anxiety (measured by the GAD-7), depression, mental health-related quality of life, and internal coherence scores. Moderate improvements were observed in sleep quality, stress, and physical health-related quality of life. In addition, the observation that most patients did not describe discomfort during the study that was possibly related to the study medication suggests a good tolerability of Bryophyllum 50% chewable tablets in this patient population. The averaged data from the entire patient population are discussed below; in a separate study, subgroup analyses will be performed to find out which patients might benefit most from the treatment (manuscript in preparation).

The primary outcome of the present study—based on the BAI score, i.e., anxiety symptoms—allows us to assess anxiety symptoms as they are perceived by patients with various diagnoses. The observed average improvement in the BAI score, from 27.4 to 22.4 and 20.6—i.e., by −5.0 and then −6.8 points—after two and three weeks of treatment, points to clinical relevance. BAI scores ≥ 26 indicate severe anxiety, whereas scores of 16–25 reflect moderate anxiety. That the average score in our study changed from severe to moderate anxiety is striking, as is the reduction in the number of patients with severe anxiety (Figure 1). Moreover, at two and three weeks of treatment, the Cohen’s d values for the decreases in BAI scores were 0.58 and 0.69, respectively, i.e., they can be considered at least medium range, corroborating a substantial reduction in anxiety symptoms. The BAI score reduction observed during our study is comparable to or stronger than those reported in other studies on herbal medicines to treat anxiety that also used BAI scores as an outcome. For example, in a study with assessments at baseline and after two weeks of treatment, much weaker reductions in the BAI score were observed in both the verum and placebo groups (−0.59 vs. −0.11 points) [49]. Moreover, studies with longer treatment periods reported comparable average improvements after treatment with verum and, in part, markedly weaker improvements in the placebo group [50,51,52].

The improvements in anxiety symptoms were captured as well by the GAD-7, a questionnaire recommended by the international, multidisciplinary working group under the leadership of the International Consortium for Health Outcomes Measurement (ICHOM) [53]. In fact, our study’s Cohen’s d values were even higher in the case of GAD-7 than in BAI. More than the GAD-7, the BAI represents an attempt to reduce overlap with depressive symptoms and focus on somatic symptoms such as heart racing and dizziness [54]. Therefore, the considerable number of patients suffering from depression could have contributed to the GAD-7 capturing somewhat better the improvement of anxiety symptoms. In fact, both reports from the patients and data from their clinical records show that the most common diagnosis group in our patient collective was depression. This corresponds well with data on depression obtained with the PHQ-9 questionnaire, which is highly recommended by the ICHOM. The PHQ-9 depression scores confirm that most patients in the present study were suffering from depression at study start: the average initial PHQ-9 depression score was 17, indicating moderately severe depression. Our results showing improvements in anxiety symptoms and pointing towards a reduction in depression severity may be of interest to numerous patients suffering from various mental and behavioral disorders. That the patients noticed improvements is corroborated by the high compliance during this study and by the frequent requests for additional Bryophyllum 50% chewable tablets for the period after the study in those cases in which the patients could not be directly admitted to the hospital. It is worth mentioning that the study design (during the waiting time) forced us to treat the patients for only 2–3 weeks, which is relatively short for patients with anxiety symptoms in the context of depression; from this perspective, the improvements appear particularly promising. A reduction in perceived stress and strong improvements in mental health-related life quality and in the field of health resources (such as coherence, inner resilience, and the thermo-coherence dimension of the ICS) were observed as well. Interestingly, these strong improvements were observed in a patient population with scores that clearly diverge from those typical for healthy persons.

Treatment of anxiety symptoms—especially in the context of depression—with synthetic medications is complex; patients often have to accept living with some marked side effects, and remissions are frequent [7,8,55]. Eventual withdrawal of synthetic medications is a therapy goal but can be approached only in due time and with caution, with the same applying for replacement with phytomedicines. Replacement by *Bryophyllum* spp. preparations might be feasible in only a minority of patients, but even a dose reduction in synthetic medications could be considered favorable (compare with [31]). *Bryophyllum* spp. preparations might be of particular interest for patients who want to reduce/stop synthetic psychoactive medications either when the standard treatment is completed or due to their side effects. The same applies to vulnerable populations, e.g., pregnant women, children, and polypharmacy patients who want to avoid synthetic psychoactive medications due to possible interactions.

The effect of Bryophyllum 50% on sleep quality has been previously investigated in prospective studies conducted among various populations. After an initial study on sleep quality during pregnancy in which some items from the PSQI questionnaire were used [28], the entire PSQI was used in two further studies of sleep quality during treatment with Bryophyllum 50% chewable tablets. One was carried out with cancer patients (improvement from 12.2 ± 3.6 to 9.1 ± 3.6 after a 3-week treatment [29]) and the other with nocturia patients (improvement from 7.7 ± 3.7 to 6.6 ± 3.4 after a 3-week treatment [30]). In the present study, the magnitude of the PSQI improvement was similar to that reported in the nocturia study and inferior to that observed in cancer patients (doses were as in the present study, but with a two-point-higher baseline value). Also in the present study, the Cohen’s d value for the effect on PSQI was lower than for all other scores, which suggests that effects of Bryophyllum 50% chewable tablets on sleep quality after two and three weeks could be derived from the stronger improvements in other aspects of mental health. A connection between effects on anxiety and sleep quality was suggested by the authors of the previous study with BP tea and zebrafish [36]. However, in this latter case, the reduction in anxiety-like behavior was observed the day after exposure to BP tea during the sleep cycle, suggesting that sleep quality improvements might be the primary effect.

Although previous studies have reported a good tolerability of Bryophyllum 50%, a few discomforts were reported by 13 out of the 54 patients of our study. The types of discomfort reported were highly varied, with no recognizable pattern. The nature of the discomfort and the patient population, which in our case was suffering from psychiatric/psychosomatic disorders, suggests somatization. Interestingly, the reported discomforts did not translate into compliance problems; in fact, the high level of patient compliance suggests good tolerability. For comparison, in the previous study on nocturia, only 8 out of 49 patients perceived discomfort, with 2 of these being classified by the authors as possibly associated with the treatment, namely, stomach pain and whole-body itching [30]. In the earlier study with cancer patients, 6 out of 20 patients reported discomfort that might have been caused by Bryophyllum 50% (fatigue *n* = 3, dry throat *n* = 1, agitation *n* = 1, difficult digestion *n* = 1). In comparison with the typical side effects of synthetic medications, these discomforts can be considered minimal.

Several of the reported pharmacological effects of extracts and some fractions of BP could at least in part explain the improvements in anxiety and depression scores observed in our study. In particular, the reduction in anxiety-like behavior observed in zebrafish fed with BP tea [36] is in line with our observations on anxiety outcomes in patients. The same applies to the CNS-depressant effects of a BP methanolic fraction observed in rats and mice [33] and the neurosedative effects of an aqueous fraction on mice [34,35], which both support effects on sleep quality and associated mental conditions. One can further speculate that the memory-enhancing effects—as reported for a methanolic extract on mice [56,57] as well as the neuroprotective properties of BP—such as those observed with flavonoids in aluminum-induced neurotoxicity in a rat model [43]—should contribute to improvements in mental conditions. The neuroprotective properties might involve, in addition to augmentation of several antioxidant systems, downregulation of acetylcholine esterase transcripts [43]. The latter appears particularly interesting, as acetylcholine esterase inhibitors seem to (at low concentrations) have antidepressant effects [58].

Various analytical studies have shown that BP leaf extracts and also press juice, the active ingredient of Bryophyllum 50% chewable tablets, comprise multiple compounds (cf. Introduction). At present, it is not possible to state which component(s) are specifically responsible for the observed clinical effects. Nevertheless, it is worth noticing that several water-based mixtures—and as such, likely enriched in constituents with hydrophilic properties—seem to be particularly active. This is the case with the whole press juice used to produce the chewable tablets from the present study, the aqueous fraction utilized to investigate neurosedative effects on mice [34,35], and the BP tea used in the zebrafish study [36]. On the other hand, some studies with fractions have shown that polyphenols, in particular flavonoids, might be particularly relevant (e.g., for the neuroprotective effects of BP leaves [43]). Finally, there is some evidence that bufadienolides might be responsible for the sedative effects [47,48].

Study strengths and limitations: Before the present study, there were no prospective clinical studies on the effects of any Bryophyllum spp. preparations on anxiety symptoms. To obtain a first estimation of the effect size of treatment with Bryophyllum 50% chewable tablets on anxiety symptoms and to find out which patients with mental and behavioral diseases would profit the most from the treatment, we chose a short-term, single-arm study design (without a control group). While the results encourage further research and will help us to plan a subsequent, randomized trial, the main limitation of our study is that it is not possible to calculate the contribution of a possible “placebo effect” to the observed improvements, and no causality between treatment and observed improvements can be proven. When asked about their expectations of symptom reduction under Bryophyllum 50% treatment, the majority of patients reported expecting at least some improvement in their symptoms; the influence of expectations will be explored elsewhere (manuscript in preparation). That the pre/postdesign allows estimating changes under real-world conditions, however, can be seen as a strength. A further strength is that this study took place during the waiting time for an instay in the hospital, a period in which (additional) medication changes hardly take place. However, it is difficult to fully estimate the influence of waiting for an instay in a psychiatric and psychosomatic department: some relief and tension both appear understandable. It should, however, be added that there were no clinical reasons to expect spontaneous improvements during the study duration: the patients had such severe symptoms that a stay in a psychiatric/psychosomatic department had been prescribed by their physicians. Finally, the relatively high dropout rate can be seen as a study limitation. However, the reasons for dropout were mostly not related to the study medication, and the observed rate is within the range of the dropout rate in clinical studies with medications used in the treatment of depression, the clinical diagnosis of most of our patients (with a usual dropout rate up of to 63%, cf. [59]).

## 4. Materials and Methods

### 4.1. Study Design and Ethical Approval

The study presented here has a prospective, open, monocentric, single-arm design (phase IV study) and was performed as an Investigator-Initiated Trial (IIT). During the three-week study, participants were treated with Bryophyllum 50% chewable tablets (see Section 4.3 for detailed information on the tablets) and filled out questionnaires in German at three different time points: at baseline (part 1), after two weeks of tablet intake (part 2), and after three weeks of tablet intake (part 3). This prospective study was conducted in accordance with the Declaration of Helsinki and Good Clinical Practice (GCP) and approved by the Ethics Committee northwest/central Switzerland (KEK Nr.: 2021-00617). This study was registered on clinical trials.gov (NCT04825171). All study participants gave written informed consent.

### 4.2. Study Patients

Participants were recruited from patients on the waiting list for the Psychiatry and Psychosomatic Departments of the Klinik Arlesheim; all diagnoses were considered. All patients from the waiting list had been referred to the Klinik Arlesheim by their treating physician (general practitioner or psychiatrist). The necessity for a waiting list is due to the high number of referring physicians who prescribe an inpatient stay at the Klinik Arlesheim. The screening took place after the initial physician–patient consultation unless it was already clear that the patient would not fulfill the inclusion criteria of the study; no additional selection of patients was conducted.

As part of the screening, the anxiety levels of the patients were assessed using the brief 2-item Generalized Anxiety Disorder questionnaire (GAD-2), on which a score of 3 points (out of 6 achievable points) was used as a cut-off for study inclusion. The 2 items were the following: how often over the last two weeks have you been bothered by the following problems: (1) feeling nervous, anxious, or on edge, and (2) not being able to stop or control worrying. Possible answers were as follows: not at all, several days, more than half the days, and nearly every day [60].

Exclusion criteria were as follows: age under 18, treatment with Bryophyllum 50% chewable tablets during the past two months, a wheat allergy, and planned dose increases or the introduction of new (conventional) anxiolytics or antidepressants for the duration of this study. Apart from the screening, no additional visits at the Klinik Arlesheim were required.

Participants completed the self-reported inventories on paper at home and were asked to return the questionnaires as well as the Bryophyllum 50% flask with the remaining tablets at the end of the study.

**Figure 3:**
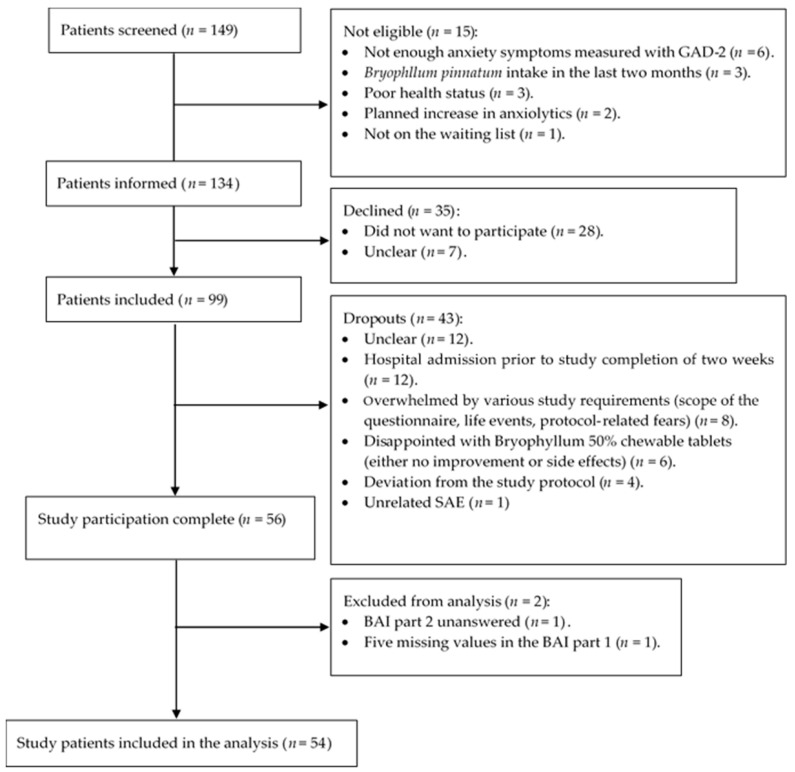
Flowchart of patient screening, recruitment, and analysis procedures.

The initial sample size determination (cf. Section 4.6) revealed that 51 patients with BAI scores at baseline and after two weeks of treatment would be necessary; therefore, screening was continued until this number was achieved. As shown in Figure 3, a total of 149 patients were screened. Of these, 15 patients turned out not to be eligible for this study, resulting in 134 patients who were informed about the study. Thirty-five of the informed patients declined participation. From a total of 99 patients included, 43 dropped out during the course of this study. For 12 patients, it was not possible to find out why they dropped out and/or did not return the completed questionnaires. Twelve patients were admitted to the Klinik Arlesheim before they could fill out the second questionnaire after two weeks of treatment; since they were no longer on the waiting list, the clinic admission was seen as an exclusion criterion.

Ten patients withdrew their patient consent because they were unable to complete this study. The following reasons were mentioned at study withdrawal: difficult personal situation (*n* = 1), because it is too much (*n* = 5), fear of lactose in the tablets (*n* = 1), and fear of the wheat contained causing rashes (*n* = 1). Six patients withdrew from this study because they were disappointed with the treatment effect: in two cases because no effect was felt and in four cases due to adverse events, namely, diarrhea (probably due to lactose, *n* = 1), trembling of the body (*n* = 1), nettle rash (*n* = 1), and stomach problems (*n* = 1). One patient was admitted to another hospital (*n* = 1, SAE).

### 4.3. Intervention

Bryophyllum 50% chewable tablets were manufactured by Weleda AG, Arlesheim, Switzerland, and were produced by drying fresh pressed leaf juice with lactose (composition of the active ingredient: 5 g leaf press juice processed in 10 g of lactose) and prepared according to good manufacturing practices (GMP). In Switzerland, they are authorized by the Swiss Agency for Therapeutic Products (Swissmedic) without medical indication. Bryophyllum 50% chewable tablets are defined based on manufacturing procedures and specifications (herbal extract of the category “other extracts” according to the European Pharmacopoeia). According to the manufacturer, these procedures and specifications comprise characteristics of the press juice (dry residue, color, and pH) and of the 50% leaf press juice and lactose granules (temperature, particle size, pH, and residual moisture content). A similar product is available as a powder in Germany, where the authorization process for the chewable tablet form is ongoing. A voucher specimen (no. ZSS29717) of the plants from which the leaves are collected has been deposited at the Zurich Succulent Plant Collection, Switzerland.

Patients were instructed to chew 3 × 2 tablets daily (two tablets at midday, two tablets in the evening, and two tablets before bedtime), i.e., the same dosage as in a previous study on overactive bladder [26]. For comparison, in a past clinical trial on acute tocolysis [25], a 4 × 2 dosage was used. In sleep studies [29,30], in which a nocturnal effect was desired, patients were instructed to take 2 tablets twice daily (with the evening meal and when going to bed).

### 4.4. Measurements

The baseline questionnaire included additional sociodemographic (age, BMI, education level, employment status) and health-related (stress factors, diseases, medication, therapies) questions. Since patient attitude towards a medical intervention is an important modulator of treatment outcome, we also asked about the patients’ expectations regarding the Bryophyllum treatment. In parts 2 and 3 of the questionnaire, participants could state any side effects and discomforts they had perceived during the treatment period. The kind of discomfort, the duration, the intensity, and countermeasures could be specified.

The Beck Anxiety Inventory (BAI) assesses four subgroups of anxiety-related symptoms occurring in the last seven days: subjective (e.g., “being terrified”), neurophysiological (e.g., “numbness or tingling”), autonomic (“indigestion”), and panic (“heart pounding”). The inventory consists of 21 questions with a 4-point Likert-type scale ranging from 0 (not at all) to 3 (severely). The BAI was used to measure the severity of anxiety, with a total score varying from 0 to 63, with higher numbers indicating more severe anxiety symptoms. BAI scores are classified as minimal anxiety (0 to 7), mild anxiety (8 to 15), moderate anxiety (16 to 25), and severe anxiety (26 to 63) [61,62]. Cases with more than three missing items were excluded in the analysis.

The Generalized Anxiety Disorder (GAD-7) scale was used to measure symptoms and severity of anxiety in the past two weeks. The scale consists of seven items rated on a 4-point Likert scale ranging from 0 to 3. Scores range from 0 to 21, with higher scores indicating more severe symptoms. Total scores classify symptoms as normal (0–4), mild (5–9), moderate (10–14), and severe (15–21) [63]. A total score ≥8 is indicative of clinically significant anxiety symptoms and of a DSM-IV diagnosis of an anxiety disorder [64]. While the original publication does not explicitly mention how to handle missing items, mean substitution of a missing value was performed if only one item was missing.

The Depression Scale (PHQ-9) was used to assess the presence and severity of depressive symptoms in the past two weeks. The scale consists of nine items with a 4-point Likert scale. Scores range from 0 to 27, with higher scores indicating more severe depressive symptoms [65,66]. A total score of 0–4 indicates no depressive symptoms, 5–9 indicates mild, 10–14 indicates moderate, 15–19 indicates moderately severe, and 20–27 indicates severe depressive symptoms [67]. This questionnaire is widely used as a diagnostic and severity inventory [68] and is able to detect changes in depression over time [69]. If more than one item was missing, no score was calculated.

The Pittsburgh Sleep Quality Index (PSQI) assesses sleep quality and comprises the following seven components, each one scored from 0 (no difficulty) to 3 (severe difficulty): subjective sleep quality, sleep latency, sleep duration, sleep efficacy, sleep disturbance, use of sleep medication, and daytime dysfunction. The total score ranges from 0 to 21, with a higher score indicating poorer sleep quality. A cut-off score of > 5 is considered as a significant sleep disturbance [70]. Substitution of missing values by estimation was not intended. If an item was missing, the questionnaire was not analyzed. Following the study design, patients were asked to rate their sleep in the last two weeks (rather than in the last four weeks).

The Perceived Stress Scale (PSS-10) measures perceived stress. The questions ask about the frequency with which someone has had to deal with certain thoughts and feelings during the last month and are rated on a 4-point Likert scale ranging from 1 (never) to 5 (very often). A total score can range from 0 to 40, with a higher score indicating a higher level of perceived stress [71,72,73]. While the original publication does not explicitly mention how to handle missing items, mean substitution of a missing value was performed if only one or two items were missing. Following the study design, patients were asked to rate their stress in the last two weeks (rather than in the last four weeks).

The Short Form of the health-related quality of life questionnaire with 12 items version 2 (SF-12) measures the health status, which can be assigned to the basic dimensions of physical health (such as physical functioning, role—physical, bodily pain, general health) and mental health (such as vitality, social functioning, role—emotional, mental health) [74]. Following the study design, patients were asked to rate their quality of life in the last week rather than in the last four weeks. According to the manual, subscales were norm-based scored using the data from the US population 1990. Substitution of missing values by estimation is not intended. If an item was missing, the score was not analyzed [75].

The relatively new Internal Coherence Scale (ICS) was used to assess internal coherence in the last week [76]. The ICS is a compact ten-item scale based on two subscales, inner resilience and coherence (eight items) and thermo-coherence (two items), with robust reliability (rα = 0.91, rrt = 0.80) and validity [76,77]. A total score ranges from 10 to 50, with inner resilience and coherence ranging from 8 to 40 and thermo-coherence from 2 to 10, with a higher score indicating higher internal coherence and resilience. Mean substitution of a missing value was accepted if only one item was missing [76].

Information on the date of hospital admission, ICD-10 codes for diagnoses at hospitalization, and whether a patient asked for a Bryophyllum 50% prescription was retrieved from the clinical information system and noted in the CRF. All data collected on paper were manually entered into the web-based data application REDCap [78,79] using the double data entry method, which includes record merging by a third person.

In order to assess how well patients adhered to the Bryophyllum 50% treatment, at the end of this study they were asked to count the remaining tablets in the flask and report it in the third questionnaire. Study personnel performed a double compliance check and counted the remaining tablets in the flasks that were sent back (44 of 54).

### 4.5. Endpoints

#### 4.5.1. Primary Endpoint

Changes in anxiety symptoms were assessed with the BAI between baseline and after two weeks of Bryophyllum 50% treatment.

#### 4.5.2. Secondary Endpoints (Except for BAI, Between Baseline and After Two or Three Weeks of Treatment)

1. Changes in anxiety symptoms assessed with the BAI between baseline and after three weeks of treatment.
2. Changes in anxiety symptoms assessed with the GAD-7 questionnaire.
3. Changes in symptoms of depression assessed with the PHQ-9.
4. Changes in PSQI.
5. Changes in PSS.
6. Changes in SF-12.
7. Changes in ICS.

### 4.6. Statistical Analyses

Before initiating this study, the sample size was determined as follows. Assuming a moderate effect on BAI after two weeks of treatment (effect size and standard deviation as in a previous paper by de Beurs et al. [80]), a Cohen’s effect size of 0.4, a significance level of 0.05, and power of 0.8, it was found that 51 patients would be needed to disprove the null hypothesis (no difference between baseline and after two weeks of treatment in a two-sided paired t-test). The screening of patients was conducted till 51 patients had sent duly filled-in BAI questionnaires at baseline and after two weeks of treatment.

Various sociodemographic and health-related variables were descriptively characterized. Data were analyzed with the statistical software package SPSS (IBM Corp. Released 2021. IBM SPSS Statistics for Windows, Version 28.0. Armonk, NY: IBM Corp). For the primary outcome, two-tailed *p* values were calculated using the Student’s t-test for paired samples. *p* values < 0.05 were considered statistically significant. Since the evaluation of the secondary outcomes is purely exploratory, other *p* values were calculated but reported without claiming significance. In each questionnaire, missing item values were treated as stated in the corresponding manual (cf. Section 4.4). For patients missing the whole third questionnaire, the last observation carried forward (LOCF) analysis was applied. The missing values were replaced by the patient’s previously collected values from the second questionnaire. The combination of the collected and imputed data was then analyzed as though there were no missing data.

## 5. Conclusions

The effect sizes observed in this prospective, single-arm study suggest that treatment with Bryophyllum 50% chewable tablets is associated with a substantial and clinically significant reduction in anxiety symptoms and improvements in related disorders. More specifically, the results show that anxiety symptoms measured with the BAI in psychiatric and psychosomatic outpatients are significantly reduced after two and three weeks of Bryophyllum 50% intake (2 × 3 chewable tablets per day, with high compliance), with mean BAI scores dropping from the category of severe anxiety to that of moderate anxiety. In addition, they reveal that marked improvements are observed in general anxiety (measured by the GAD-7), depression, mental health-related quality of life, and internal coherence scores, whereas moderate improvements are observed in sleep quality, stress, and physical health-related quality of life. Taken together, the findings are in line with previous pharmacological investigations and support the use of Bryophyllum 50% chewable tablets as an effective and well-tolerated treatment option for patients with anxiety symptoms. Since the study design does not allow for conclusions on the causal relationship between treatment and observed improvements, a randomized clinical trial is urgently needed. To better define the groups of patients most appropriate for such a study, subgroup analyses of the present patient collective should be performed.

## Figures and Tables

**Table 1:** Sociodemographic characteristics of the study participants.

	*n* or Mean ± SD	Percent	Median (Max–Min)
Age (*n* = 53) ^a^	51.0 ± 13.9		53.0 (83–25)
BMI (*n* = 52)	23.5 ± 4.8		22.0 (34.8–16.9)
Gender (*n* = 52)			
Woman	41	78.8	
Man	10	19.2	
Other	1	2.0	
Highest level of education (*n* = 51)			
Compulsory school	4	7.8	
Apprenticeship	14	27.5	
High school	6	11.8	
Higher education	10	19.6	
Tertiary education (university and others)	17	33.3	
Living situation (*n* = 59) ^b^			
Alone	30	50.8	
With partner	14	23.7	
With partner and child/children	7	11.9	
Other	8	13.6	
Employment (*n* = 33) ^c^			
0–20%	4	12.1	
21–40%	6	18.2	
41–60%	5	15.2	
61–80%	7	21.2	
81–100%	11	33.3	
Nicotine consumption (*n* = 46)			
Smoker	15	32.6	
Sports (*n* = 54)	32	59.3	

^a^ Number of patients who responded is shown in brackets. ^b^ A total of 59 answers given by 54 patients, where 5 patients reported two different living situations. ^c^ A total of 21 patients did not answer this question; of these, 14 were of working age.

**Table 2:** Self-reported health-related characteristics as reported by the patients at baseline and clinical diagnoses.

Characteristic (Total Number of Answers)	Number of Patients (Answered Yes)
Self-reported diabetes (*n* = 43)	2
Self-reported high blood pressure (*n* = 48)	7
Self-reported allergies (*n* = 19)	32
Other self-reported physical illness (*n* = 16)	16 ^a^
Self-reported sleep problems (*n* = 52)	44
Self-reported anxiety symptoms (*n* = 48)	48 ^b^
Self-reported anxiety disorder (*n* = 44)	38
Self-reported depression disorder (*n* = 43)	43
ICD-10 main diagnosis ^c^ (*n* = 49)	
Moderate depressive episode (F32.1)	11
Severe depressive episode without psychotic symptoms (F32.2)	3
Premenstrual dysphoric disorder (F32.81)	1
Recurrent depressive disorder, current episode moderate (F33.1)	20
Recurrent depressive disorder, current episode severe without psychotic symptoms (F33.2)	10
Panic disorder (episodic paroxysmal anxiety) (F41.0)	2
Predominantly obsessional thoughts or ruminations: obsessive–compulsive disorder (F42.0)	1
Emotionally unstable personality disorder: borderline (F60.31)	1
ICD-10 secondary psychiatric diagnosis ^c^ (*n* = 32)	
Mental and behavioral disorders due to harmful psychoactive substance use (F10.1)	1
Mental and behavioral disorders due to use of sedatives or hypnotics: dependence syndrome (F13.2)	1
Bipolar affective disorder (F31)	1
Recurrent depressive disorder, current episode moderate and severe (F33.1 and F33.2)	3
Social phobias (F40.1)	2
Panic disorder (episodic paroxysmal anxiety) (F41.0)	5
Generalized anxiety disorder (F41.1)	2
Predominantly compulsive acts: obsessive–compulsive disorder (F42.1)	1
Post-traumatic stress disorder and adjustment disorders (F43.1 and F43.2)	6
Somatoform disorders (F45.0, F45.32, and F45.41)	4
Insomnia (F51.0)	1
Personality disorders (F60.3 and F60.5)	2
Mixed and other personality disorders (F61.0)	2
Habit and impulse disorders (F63)	1

^a^ The following other self-reported physical illnesses were reported in the questionnaire (*n* = 1 or 2 each), 24 in total: arthrosis, chronic reflux, endometriosis, gastritis type A, skin problems, long COVID, lynch syndrome, migraine, hypothyroidism, carpal tunnel syndrome, PMDS, sarcoidosis, thrombosis, and fatigue fractures. ^b^ The 48 patients reported 75 symptoms. ^c^ ICD-10 diagnoses (main and secondary) were extracted from the clinical information system; presumptive diagnoses were not included.

**Table 3:** Self-reported medications ^a^ and therapies during the study and one month prior.

Medications/Therapies	Number of Patients (Number of Medications/Therapies)
Antidepressant medications	
Antidepressants	26 (30)
Herbal medications	12 (12)
Anxiety-reducing medications	
Anxiolytics	10 (11)
Antidepressants	23 (25)
Herbal medications	6 (6)
Soporific medications	
Anxiolytics	7 (8)
Antidepressants	11 (11)
Neuroleptics	4 (4)
Herbal medications	16 (16)
Analgesic medications	9 (16)
Integrative medications and supplements	
Homeopathic preparations	6 (10)
Herbal drugs for other indications	4 (4)
Supplements	15 (34)
Self-reported therapies	
Talking therapies (*n* = 33)	33 (36)
Complementary therapies (*n* = 13)	13 (17)

^a^ Medication classes are shown as reported by the patients and classified by the authors (see text).

**Table 4:** Anxiety symptoms (BAI) before and after treatment with Bryophyllum 50% chewable tablets.

	Baseline	Two Weeks’ Treatment			Three Weeks’ Treatment ^a^		
	Mean ± SD	Mean ± SD	*p* Value	Cohen’s d	Mean ± SD	*p* Value	Cohen’s d
BAI score (*n* = 54)	27.4 ± 12.0	22.4 ± 12.1	<0.001	0.58	20.6 ± 12.9	<0.001	0.69
Factor 1: Cognitive	11.3 ± 4.5	9.1 ± 4.7	<0.001	0.57	8.6 ± 4.9	<0.001	0.67
Factor 2. Neuromotor	8.6 ± 4.4	7.3 ± 4.9	0.009	0.37	6.6 ± 5.0	<0.001	0.52
Factor 3: Autonomic	4.4 ± 2.6	3.7 ±2.5	0.009	0.37	3.4 ± 2.7	0.002	0.45
Factor 4: Panic	3.1 ± 2.6	2.3 ± 2.1	0.002	0.45	2.0 ± 2.6	<0.001	0.61

^a^ Six answers were corrected with LOCF for part 3.

**Table 5:** Changes in generalized anxiety (GAD-7), depression (PHQ-9), sleep quality (PSQI), stress (PSS), health-related quality of life (SF-12), and internal coherence (ICS) during treatment with Bryophyllum 50% chewable tablets.

	Baseline	Two Weeks’ Treatment			Three Weeks’ Treatment		
	Mean ± SD	Mean ± SD	*p* Value	Cohen’s d	Mean ± SD	*p* Value	Cohen’s d
GAD-7 score (*n* = 54)	13.6 ± 4.8	10.4 ± 5.6	<0.001	0.77	10.1 ^a^ ± 5.7	<0.001	0.76
PHQ-9 score (*n* = 54)	17.0 ± 5.3	14.2 ± 6.2	<0.001	0.58	12.7 ^a^ ± 6.6	<0.001	0.67
PSQI (*n* = 48)	10.3 ± 3.3	9.2 ± 4.0	0.004	0.44	9.1 ^a^ ± 4.2	0.006	0.42
PSS score (*n* = 54)	21.1 ± 4.2	19.0 ± 4.2	<0.001	0.51	18.0 ^a^ ± 5.1	<0.001	0.63
SF-12 PH ^b^ score (*n* = 48)	40.2 ± 10.0	41.5 ± 9.8	0.219	−0.18	43.2 ^a^ ± 11.8	0.041	−0.30
SF-12 MH ^c^ score (*n* = 48)	27.2 ± 7.2	33.2 ± 9.1	<0.001	−0.76	33.6 ^a^ ± 10.8	<0.001	−0.65
ICS score (*n* = 54)	26.0 ± 6.1	29.0 ± 6.6	<0.001	−0.52	30.5 ^a^ ± 7.6	<0.001	−0.71
Inner resilience and coherence (*n* = 54)	19.4 ± 5.3	21.8 ± 5.7	0.001	−0.46	22.8 ^a^ ± 6.6	<0.001	−0.61
Thermo-coherence (*n* = 54)	6.6 ± 2.0	7.2 ± 1.9	0.002	−0.45	7.6 ^a^ ± 1.9	<0.001	−0.61

^a^ Six answers were corrected with LOCF for part 3. ^b^ PH = SF-12 physical health score. ^c^ MH = SF-12 mental health score.

**Table 6:** Intake of Bryophyllum 50% chewable tablets, changes in the doses of anxiolytics/antidepressants during the study, and possible hospital stay after the study.

Characteristic (Number of Patient Answers After Two and Three Weeks)	Number of Patients or Mean ± SD After Two Weeks	Number of Patients or Mean ± SD After Three Weeks
Tablet intake (n.a., *n* = 50)	n.a.	118 ^a^ ± 16
Daily dose of Bryophyllum 50% (*n* = 47, *n* = 41)	5.9	5.9
Bryophyllum 50% prescription requested (n.a., *n* = 54)	n.a.	32
Dosage of BP changed (*n* = 48, *n* = 43)		
Dosage not changed	41	37
Intake stopped	2	1
Dosage reduced	1	2
Intake forgotten	4	3
Dose increase in anxiolytics/antidepressants during the study (*n* = 42)	1 ^b^	2 ^c^ (4 products)
Dose decrease in medications during the study (*n* = 37)	7 ^d^	1 ^e^
Hospital admission after study end (*n* = 54)	n.a.	38

^a^ Eight patients reported taking more than 132 tablets (corresponding to 6 tablets for 22 days) or returned an empty flask. In those cases, the number of tablets taken was replaced by 132. However, if the patients returned an empty flask but reported some remaining tablets in the flask, the value reported by the patient was used to calculate the chewable tablet intake during the study. ^b^ One patient increased their dose of cannabidiol during the first two weeks of the study. ^c^ Doses of bromazepam (*n* = 1), zolpidem (*n* = 1), escitalopram (*n* = 1), mirtazapine (*n* = 1) were reported to be increased during the third week of the study. ^d^ The doses of the following medications (including herbal), most with anxiolytic/antidepressant effects, were reported to be decreased during the first two weeks of the study: a product with dry extract of valerian radix and dry extract of hops (*n* = 1), St John’s Wort (*n* = 1), mirtazapine (*n* = 1), cannabidiol (*n* = 1), a product with valerian, purple passionflower, lemon balm, and butterbur (*n* = 1), quetiapine (*n* = 1), and a product with gold and hyoscyamus (*n* = 1). ^e^ The dose of lisdexamfetamine (*n* = 1) was reported to be decreased during the third week of the study. n.a., not applicable.

## Data Availability

The datasets presented in this article are not readily available because data sharing and additional analysis would need an additional ethics permit. The authors would, however, support other authors in obtaining an ethics permit to reuse the data.
